# Supplementary material for: Porous Nb2O5 Formed by Anodic Oxidation as the Sulfur Host for Enhanced Performance Lithium-Sulfur Batteries
Source: Nanomaterials (Basel). 2023 Feb 20;13(4):777. doi: 10.3390/nano13040777 (PMC9963548; doi:10.3390/nano13040777)
Supplement: Supplementary file 1 [file nanomaterials-13-00777-s001.zip › nanomaterials-2191650-supplementary.pdf]

Supplementary Materials

Porous Nb_2O_5 Formed by Anodic Oxidation as the Sulfur Host for Enhanced Performance Lithium-Sulfur Batteries

Jianming Wang [1], Lu Chen [1], Bo Zhao [1], Chunyong Liang [1*], Hongshui Wang [1*], Yongguang Zhang [1*]

School of Materials Science and Engineering, Hebei University of Technology, Tianjin 300130, China

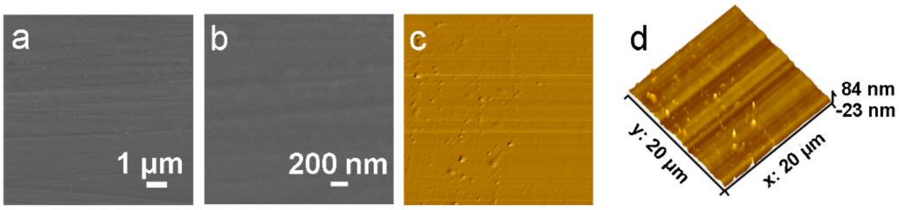

Figure S1. (a, b) SEM image of unoxidized niobium flakes; **(c, d)** AFM diagram of unoxidized niobium flakes.

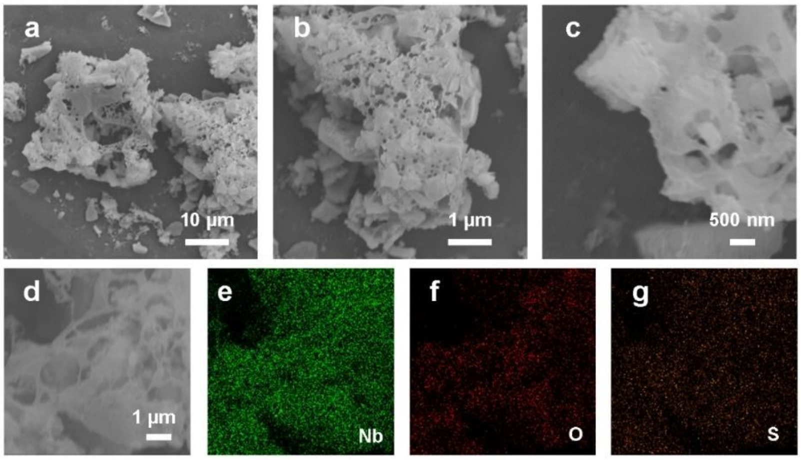

Figure S2. (a-c) SEM image of porous niobium oxide after sulphury mixing; **(d-g)** SEM images and elemental mapping images of P-Nb_2O_5 after sulphury mixing. Nb, O and S are represented in green, red and yellow, respectively.

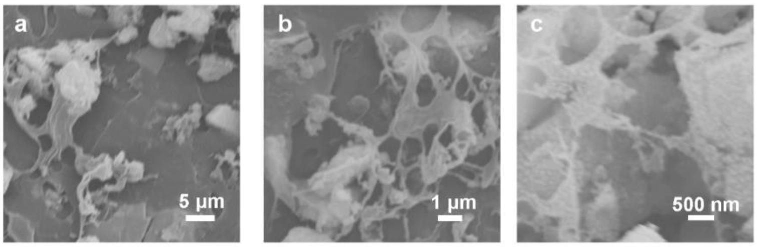

Figure S3. (a-c) SEM image of P-Nb_2O_5 electrode after cycling.

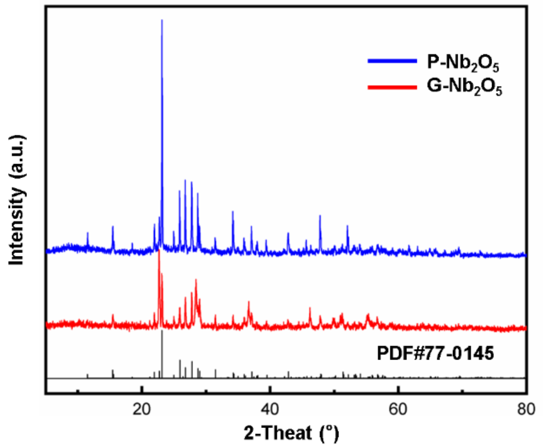

Figure S4. XRD patterns of P-Nb_2O_5 and G-Nb_2O_5 after sulphury mixing.

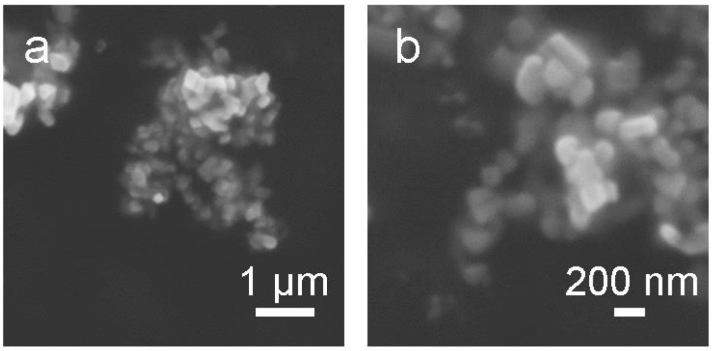

Figure S5. (a, b) SEM image of G-Nb_2O_5.
